# Supplementary material for: Unraveling the Role of Azobenzene‐Based Photoswitchable Lipids in Controlling Innate Immune Response
Source: Chemistry. 2025 Nov 4;31(69):e01824. doi: 10.1002/chem.202501824 (PMC12699183; doi:10.1002/chem.202501824)
Supplement: Supplementary file 1 — Supporting Information [file CHEM-31-e01824-s001.pdf]

Supplementary Information for:

Unraveling the Role of Azobenzene-Based Photoswitchable Lipids in Controlling Innate Immune Response

Anastasiia Delova,[a] Aline M*akhloutah*,[b] *Yann Bernhard,*[b] *Andreea Pasc,*[b] *Antonio Monari.*[a,*]

a) Université Paris Cité and CNRS, ITODYS, F-75006 Paris, France. b) Université de Lorraine and CNRS, UMR 7053 L2CM, Nancy, France

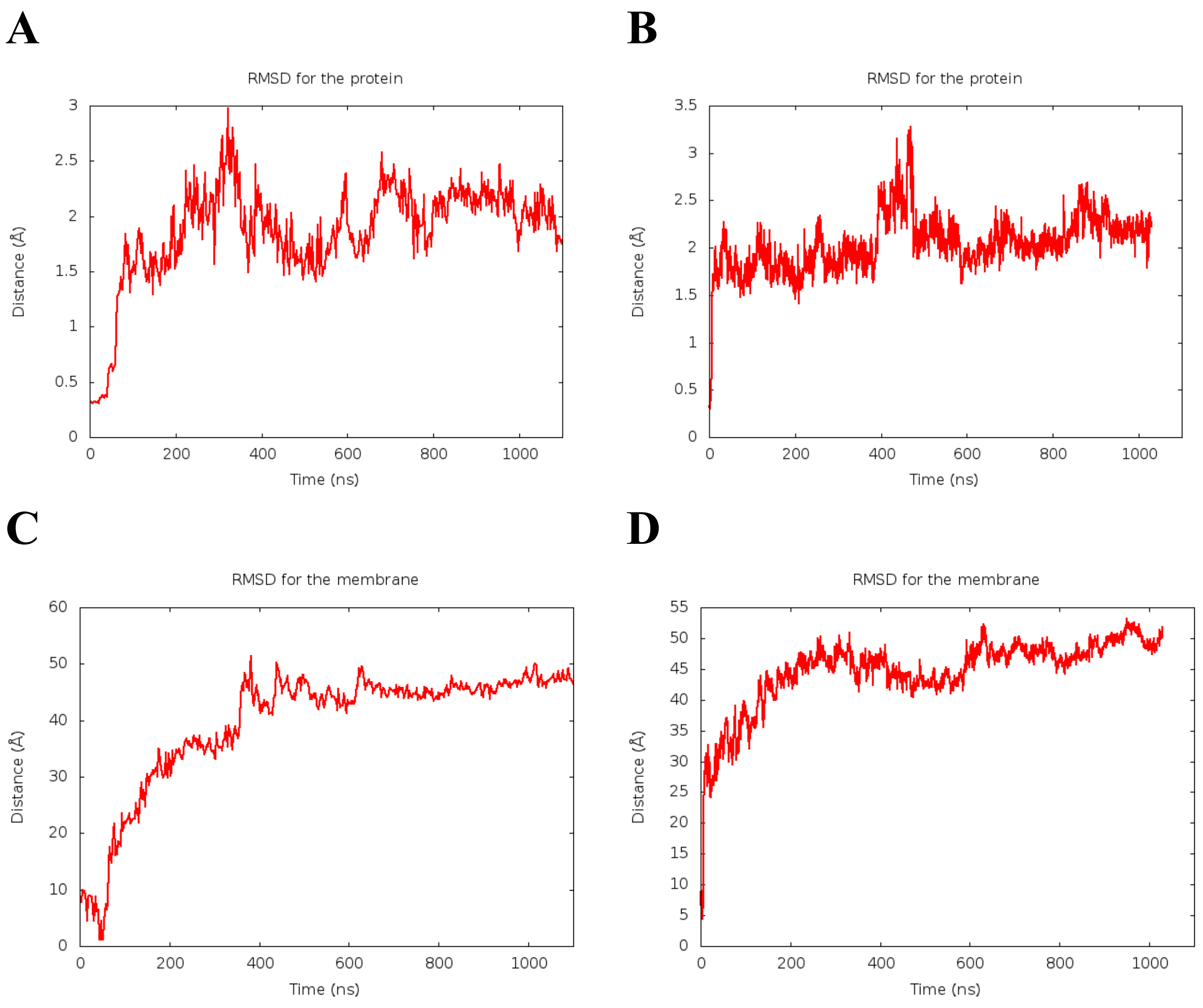

Figure S 1. Time evolution of the RMSD for IvG domain interacting with E-PSL (A) and Z-PSL (B). The RMSD for the lipid bilayer when IvG is interacting with E-PSL (C) and Z-PSL (D) are also provided.

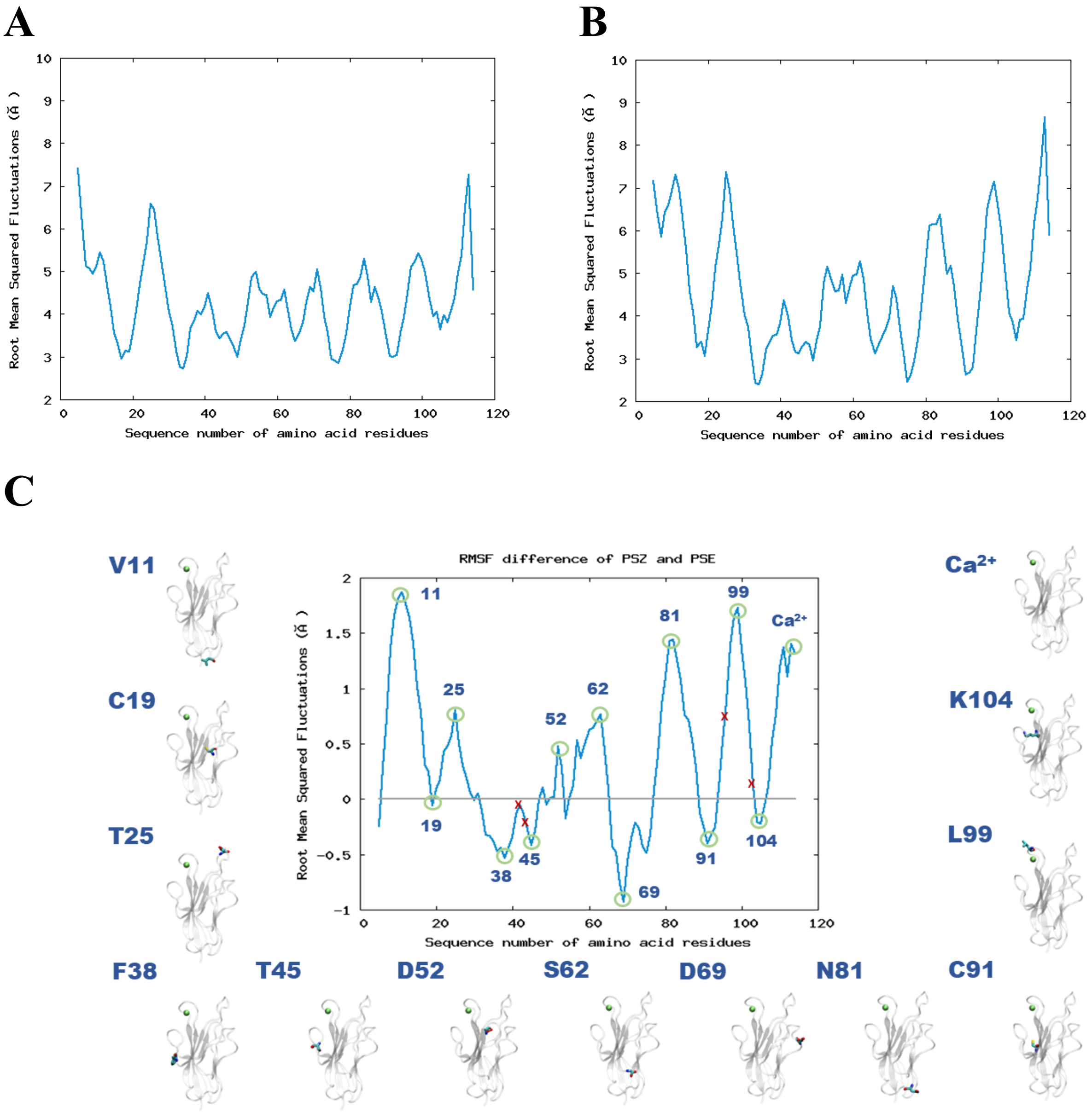

Figure S2. Per-residue RMSF for the IgV domain interacting with E-PSL (A) and Z-PSL (B), as well as the difference in RMSF, calculated as the RMSF of the protein interacting with Z-PSL minus that of the protein interacting with E-PSL (C), are presented. Positive values indicate greater flexibility in the Z-PSL-bound system, while negative values indicate increased flexibility in the E-PSL-bound system. Red crosses mark key protein residues in the binding pockets (S42, Q43, Q95, D102). Snapshots showing the position of residues experiencing the largest changes in RMSF are also provided.

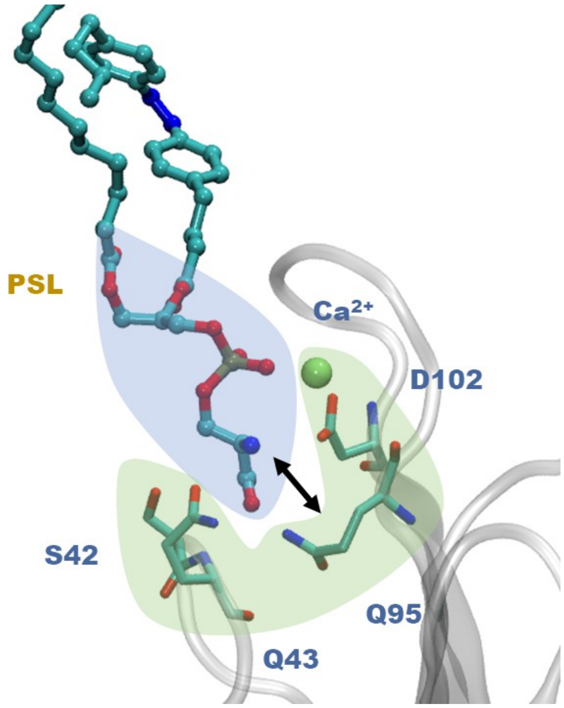

Figure S3. Representation of the collective variable used for the US calculation. The coordinate comprise he distance between the lipid polar heads (the involved atoms are highlighted in purple) and the interaction pocket of the IvG domain (highlighted in green) encompassing the Ca^{2+} ion and the residues S42, Q43, Q95, and D102.

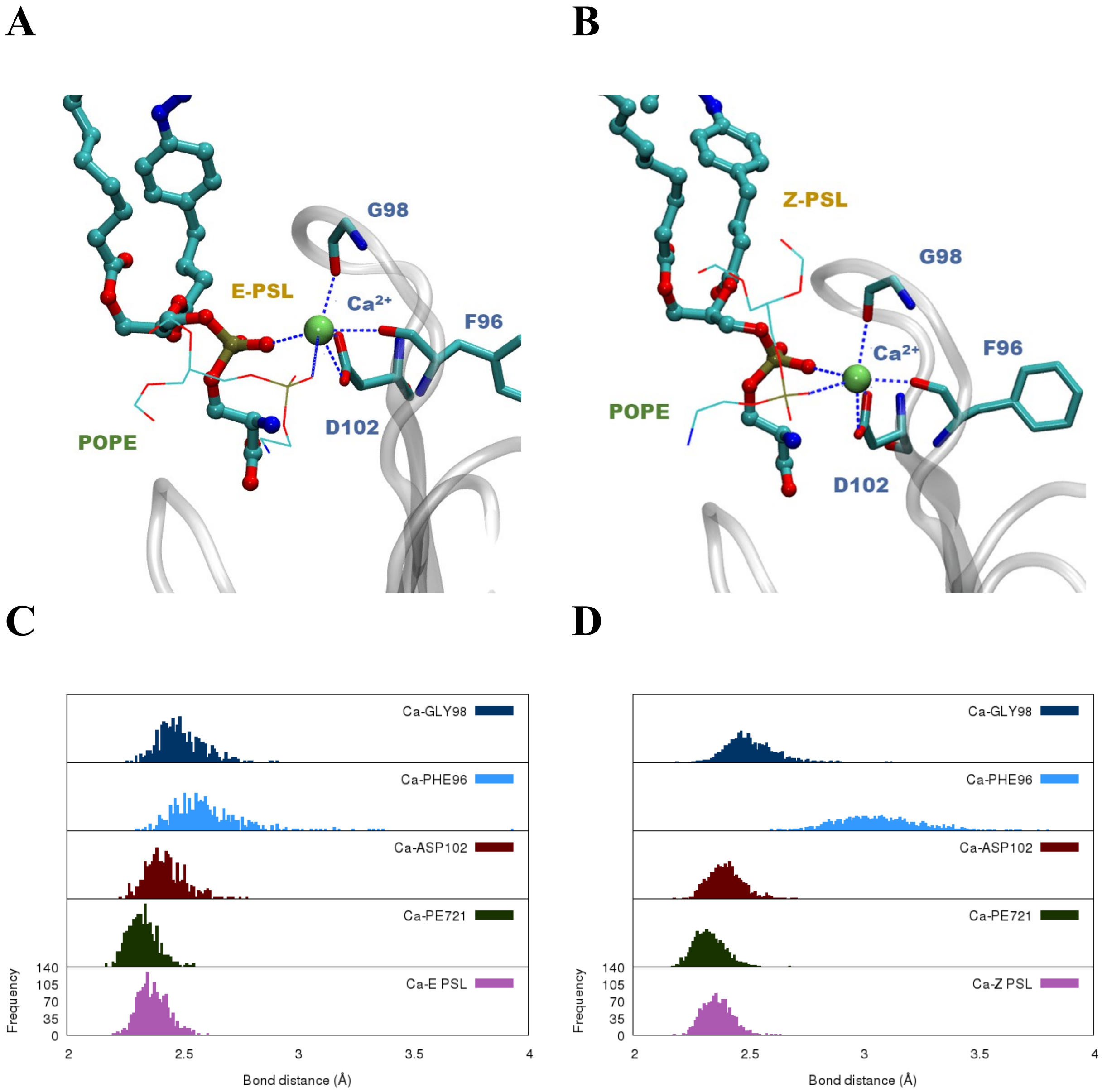

Figure S4. Representative snapshot showing the interactions developed by Ca^{2+} with the IvG residues (F96, G98, D102), PSL molecule (colored in licorice) and POPE lipid (colored in lines mode) for the systems containing E-PSL (A) and Z-PSL (B), respectively. The distribution of the crucial distances between Ca^{2+} and the protein residues are given for E-PSL (C) and Z-PSL (D).

Ca^{2+}	Native PDS (Crystal)	E-PSL	Z-PSL
PSL	2.938	2.38 ± 0.06	2.37 ± 0.06
G98	2.711	2.50 ± 0.09	2.52 ± 0.13
F96	2.578	2.61 ± 0.17	3.06 ± 0.18
D102	3.015	2.46 ± 0.32	2.46 ± 0.11
PE721	-	2.33 ± 0.65	2.34 ± 0.67

Table S1. Average values and standard deviations of the distances between Ca^{2+} ion and the principal residues of the IvG stabilizing the ion in the binding pocket. For E- and Z-PSL, conformers. The value for the crystal structure comprising an isolated PDS unit interacting with IgV are also given as a reference.

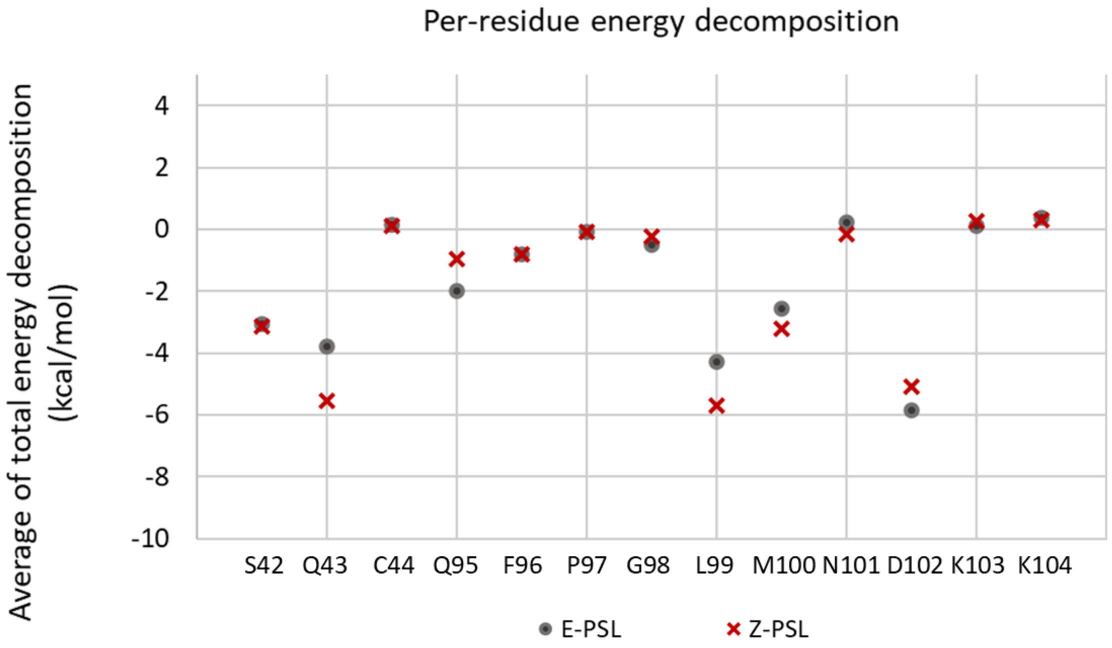

Figure S5. Per residue MMPBSA contributions.

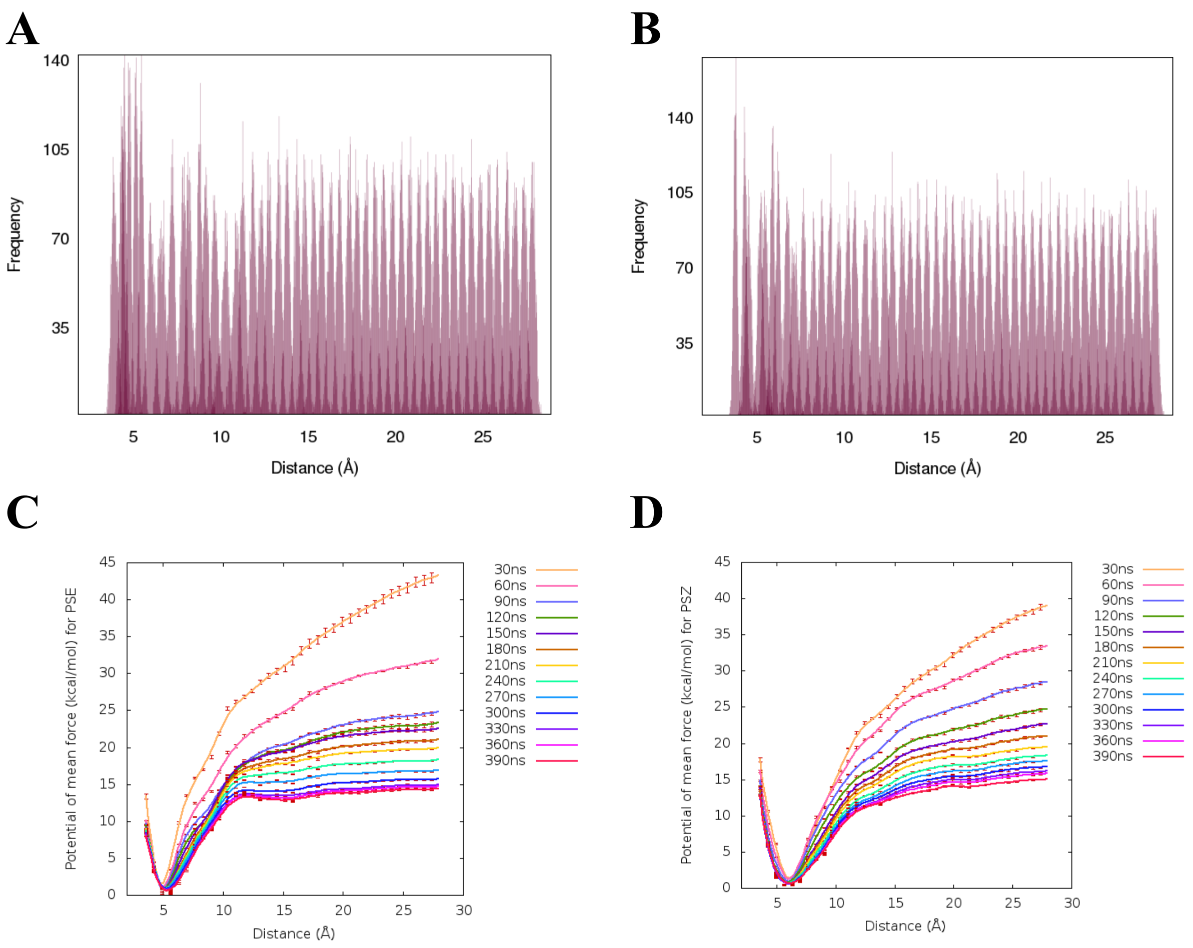

Figure S6. Distribution of the evolution of the collective variable around the different umbrella sampling for the E-PSL (A) and Z-PSL (B) conformer showing the overlap between the windows. Evaluation of the PMF convergence for E-PSL (C) and Z-PSL (D).

Entity:		Force constant	E	Z
Bonds (nm):	NN	1.40×10^7	0.1270	0.1255
	CN	0.72×10^7	0.1425	0.1440
Angles (deg):	CNN	650.0	114.0	119.0
	CCN	560.0	120.0	120.0
Dihedrals (deg):	CNNC	70.0	180	180
	CCNN	6.0	180	180

Table S2. Force field parameters used to enforce the correct geometry around the isomerizable CNNC bond as taken by Osella et al. (J. Phys. Chem. C 2020, 124, 8310–8322).
